# Children’s psychosocial wellbeing in the context of HIV/AIDS and poverty: a comparative investigation of orphaned and non-orphaned children living in South Africa

**DOI:** 10.1186/1471-2458-14-615

**Published:** 2014-06-18

**Authors:** Kaymarlin Govender, Candice Reardon, Tim Quinlan, Gavin George

**Affiliations:** 1 Health Economics and HIV and AIDS Research Division, University of KwaZulu-Natal, KwaZulu-Natal, South Africa; 2 School of Applied Human Sciences, College of Humanities, University of KwaZulu-Natal, KwaZulu-Natal, South Africa

**Keywords:** Children, Orphans, HIV/AIDS, Anxiety, Depression, Resilience, Poverty

## Abstract

**Background:**

Recent studies have questioned whether orphanhood is primarily associated with key dimensions of psycho-social wellbeing in children living in circumstances of material deprivation and high prevalence of HIV and AIDS.

**Methods:**

This study uses cross-sectional data from a longitudinal study conducted between 2004-2007 to examine the psychosocial well-being of orphans and non-orphans in the Amajuba District of KwaZulu-Natal, South Africa. Psychosocial wellbeing included an assessment of orphans’ and non orphans’ level of anxiety and depression, affability and resilience. Stratified cluster sampling, based on both school and age, was used to construct a cohort of recent orphans and non-orphans and their households, randomly selected from schools.

**Results:**

Levels of anxiety and depression, affability and resilience did not differ significantly between orphans and non-orphans, nor did salient household, poverty and caregiver characteristics vary substantially amongst orphans and non-orphans. Multivariate analyses indicated that children’s psychosocial outcomes, when controlling for orphan status and related demographic variables were more strongly influenced by household composition/size, living above or below the poverty threshold and factors associated with the caregiver-child relationship and caregiver health.

**Conclusions:**

The results muster additional evidence for moving beyond narrow definitions of vulnerability associated exclusively with orphanhood to consider the multitude of material, social and relational factors affecting the psycho-social well-being of children in general who are living in circumstances of poverty and HIV and AIDS.

## Background

There is a growing body of research on the effects of orphaning on the psychosocial and material wellbeing of children living in the context of HIV and AIDS. Results to date have not shown a consistent relationship. Some studies have shown diminished psychosocial [1-4] and material wellbeing [5] amongst ‘AIDS orphans’ compared to other children. Reported findings span a range of overlapping consequences of orphaning such as higher school dropout, more food insecurity, greater likelihood of residence in ‘poor’ households and higher (self-rated) levels of poor physical health and psychosocial distress [5-11]. In contrast, there are studies which question whether orphaning is the primary variable related to the psychosocial and material outcomes. Bhargava [12] and Cluver and Gardner [6] reported no significant differences in outcomes of orphans compared to other children. A review of 60 nationally representative household surveys from 36 different countries found that children living in households with low income were most at risk of poor psychosocial and/or material outcomes [13]. Similarly, another review [14] asserted that controlled studies point to a much more complex picture on AIDS orphan children, with data suggesting negative effects or no differences alongside some evidence of protective effects due to the quality of care and economic assistance after the death of parent(s). In terms of the latter, it is worthwhile to note the commonness of caregiving roles adopted by those other than the biological parents in the context of a long history of economic migrancy in southern Africa, including South Africa

Given the equivocal nature of findings in outcomes of orphan and non orphan children, a key aim of this study was to assess the relative influence of child characteristics (particularly orphan status), caregiver characteristics, household characteristics and material poverty indices on three child psychosocial outcomes. More specifically, depression and anxiety were used as measures of psychological distress, while resilience and affability were seen as protective factors in orphan and non-orphaned children living amidst a rampant HIV epidemic. Furthermore, we also note that there is a significant gap in this body of literature which considers the variable role of the different factors in moderating or compounding threats to the psychosocial wellbeing of children. We refer here to the child-clinical and paediatric psychology literature within which there is longstanding recognition of the complex relationship between stresses on children’s psychological states and adjustments to those stresses [15-17]. We also acknowledge the point made by Sherr et al., [14] that we need to move beyond a deficit model of child psychosocial wellbeing to also understand protective factors where children rally. Therefore, an ancillary aim of this study was to explore protective factors in different conditions of material poverty and its relation to psychosocial outcomes.

The paper is based on a longitudinal project, the Amajuba Child Health and Wellbeing Research Project (ACHWRP), which was conducted in the KwaZulu-Natal province of South Africa between 2003 and 2009. This research stemmed from concern in the early 2000s about the number of children left orphaned by the HIV epidemic [18-22]. At the time, there was little systemic research in South Africa; the anecdotal record was that there were a million ‘AIDS orphans’ in South Africa. Subsequently, there have been systemic government and civil society interventions, backed by international funding, as accrued evidence indicated higher than projected numbers of children who had lost one or both parents - approximately 3.8 million children by 2008 [23] which is still a commonly used estimate [24]. The study focused on a cohort of 623 children, consisting of a matched sample of orphan and ‘non-orphan’ children and included three surveys on these children and their caregivers, conducted at 12-15 month intervals. This paper draws upon cross-sectional data on the well-being of children from the third and final survey of the study conducted in 2006 and 2007 which included 266 orphans and 397 ‘non-orphans’.

## Method

### Study site and population

The Amajuba district incorporates the municipalities of Newcastle, Dannhauser and eMadlangeni with a population of just under half a million inhabitants [25]. There are high levels of unemployment due to the decline in the coal industry [26]. HIV antenatal prevalence in the Amajuba district has fallen from 46% in 2006 (15 to 49 year old pregnant women being tested positive for HIV in antenatal clinics) to 35.3% in 2011 [27]. The study population consisted of 623 Zulu and English speaking school-going children, between 9 - 15 years in 2004, and their caregivers and/or heads of household. The lower age limit was dictated by consideration of cognitive ability of children to understand explanations of the study and the principle of voluntary participation, including the right to refuse to answer questions and to withdraw from the study. The upper limit was dictated by need to ensure that no child was 18 years or older by the end of the survey period; 18 years old being the legal age of adulthood in South Africa. The surveys worked with three categories of households: ‘orphan households’ (those that contained orphans only), ‘mixed households’ (those containing orphans and non-orphans) and ‘non-orphan households’ (those containing no orphaned children). ‘Caregiver’ was defined as the adult in the household who primarily cared for the child participant and was not necessarily a biological parent. The sample in the 2006/7 survey round consisted of 397 non-orphaned and 226 orphaned children who were sourced from 153 ‘orphan households’, 134 ‘mixed households’ and 336 ‘non-orphan households’.

### Sampling

Random stratified cluster sampling, based on age and on school registration was used to construct a cohort of incident orphans and non-orphans and their households from a random selection of 60 schools from the 252 schools in the Amajuba District [see [28]. Incident orphans were defined as those who had lost one or both parents (irrespective of cause) within 3 months prior to the period of sample selection in 2004. Comparison (non-orphan) children were selected from the same school by grade and age group. Selection involved first, at each school, discussions with school principals and teachers to identify to identify incident ‘orphans’ within the relevant age range and ‘non-orphans’ of comparable age and gender (in the same classes as the orphans). Thereafter, there were interactive exercises which included the researchers, teachers and children in the relevant classes, to recruit in principle the orphans and non-orphans. The exercises did not reveal why some children were selected and not others, and did not draw attention to the orphan status as a reason for selection. At this point, the selected children were potential participants. The next step involved visiting the parents or guardians of each child to request their and their children’s participation in the study and to secure written informed consent from both sets of individuals. In addition, in cases where a child did not reside with his/her parent or guardian but with another adult (e.g. aunt, grandparent) who was that child’s primary caregiver, then informed consent was also obtained from that adult. The informed consent procedure was conducted in Zulu, the first language of the caregivers and children.

The survey had four components: a household and demographic information questionnaire administered to the household respondent (most often the child’s primary caregiver), a questionnaire for the primary caregiver of the study child, and two questionnaires administered to each child. Data collection involved a team of 12-14 young men and women data collectors who lived in the area and who were trained to conduct the surveys. Two-person teams (a man and a woman) would visit participants’ homes and conduct the interviews over a period of time according to arrangements made with the participants. Completed questionnaires were checked upon completion by a researcher acting as a field supervisor and, when necessary, missing or ambivalent information was noted and the data collectors would return to the homes rectify the problem. Ethical approval for the study was provided by the Boston University Medical Center Institutional Review Board and the University of KwaZulu-Natal Ethics Committee. The study was conducted in accordance with the ethical standards laid down in the 1964 Declaration of Helsinki and its later amendments.

### Measures

This paper is based on Round 3 of the data collection conducted in 2006/7. Orphans at Round 3 were classified into ‘maternal’, ‘paternal’ or ‘double orphans’ depending on whether their biological mother, biological father or both biological parents had passed away. Household type included ‘orphan only’, ‘non-orphan only’ or ‘mixed’ households of orphans and non-orphans. Poverty indicators were based on household income. ‘Monthly household income’ was a continuous variable that included any social grants received and is represented in South African Rands (ZAR). The exchange rate that was used in was 1 US Dollar (USD) = 7.260 ZAR. Household income was used to measure poverty and is represented in USD, calculated at the above exchange rate. To make comparisons across countries, the World Bank has set a common poverty threshold of USD1.25 per day which equals to USD37.5 per capita per month [29]. This threshold was used to create the categorical variable ‘poverty threshold’ that included households living below the poverty line and those living above the poverty line.

Household characteristics such as total number of inhabitants, total adults and total number of children were originally captured as continuous variables and later transformed into discrete categories in order to conduct two way ANOVA analyses. Dependency ratio represented the number of children in the household the primary caregiver looked after and was constructed as a discrete variable in the analysis. Communication with caregiver about problems was assessed with the question, “Do you talk to your parent/caregiver about your personal problems?” where response options included ‘never’ (=1), ‘sometimes’ (=2) or ‘always’ (=3). “Is your parent/caregiver helpful when you need help, money or things?” (never = 1, always = 3) was used to determine perceived ‘caregiver provision of help and assistance’. Caregivers’ health was self-assessed using the options ‘poor’ (=1), ‘fair’ (=2), ‘good’ (=3) or ‘excellent’ (=4). The level of caregiver impairment was established by caregivers answering ‘no’ (=1) or ‘yes’ (=2) to the question, “In the past 30 days were you unable to perform your most important activity for an entire day because of problems with your physical or mental health?”

### Anxiety and depression

The researchers used subscales of the Achenbach Youth Self Report (YSR) to measure anxiety/depression. The YSR has been adapted for use in populations of Zulu-speaking children and their caregivers in South Africa [30]. The final measure contained 21 items that measured anxiety/depression, Sample items included: (1) “You feel happy” and (2) “You feel like talking to other children”. Each item in the battery of questions included four response options: (1) never the case, (2) sometimes the case, (3) often the case and (4) always the case. Each subscale was scored in such a manner that a high overall total equated to more of that particular construct. The anxiety/depression subscale had an alpha reliability score of 0.87.

### Affability and resilience

Subscales of South African Child Assessment Schedule (SACAS) were used to measure affability, and resilience. Like the YRS, the SACAS has also been adapted for use in populations of Zulu-speaking children and their caregivers in South Africa [31]. The SACAS scale uses 135 items from the Child Behavior Checklist which asks the child’s caregiver about his/her skills and behavioral problems. The researchers modified this measure, asking children themselves to answer the questions and also used a much-reduced inventory with the goal of reducing burden on the child respondents given that other questionnaires in the household battery were time consuming. The final measure contained 11 items that measured affability and 7 items that assessed resilience. The affability subscale achieved an alpha coefficient of 0.76 and the resilience subscale and alpha coefficient of 0.74.

### Data analysis

SPSS version 20 was used to produce initial descriptive statistics for the variables related to adolescent characteristics, poverty related indicators, household composition, caregiver characteristics and psychosocial outcomes. Bivariate analyses (Chi square tests and t tests) were then conducted in order to examine the relationship between the variables above as a function of orphan status. A 5% level of significance was adopted as a cut-off point in each analysis. A series of two way ANOVAs, were conducted to determine if orphan status interacts with any of the four categories of variables (poverty, household, caregiver and child characteristics) to produce variation in anxiety/depression, affability or resilience scores. Post hoc tests (Scheffe test) were used to determine sources of significance in each analysis.

In the second phase of the analyses, a series of fixed effects hierarchical regression analyses were conducted to construct three models, namely, anxiety/depression, affability and resilience to explore the unique contribution of each of these categories of variables on children’s psychosocial outcomes. The tolerance values for each of these predictor variables were examined for the presence of multi-colinearity and were found to be above the level 0.10 [30]. In each analysis, we noted for significance of model effect as well as independent variables that made a unique statistically significant contribution to the model.

In these series of analyses, we also explored potential mediating and moderating relationships. The results of the bivariate and multivariate analyses suggested that a moderating relationship may be present. According to Baron and Kenny [32], moderating variables should be investigated in situations where a predictor variable is producing a weak and inconsistent association with an outcome variable.

## Results

### Descriptive statistics and bivariate analyses

Descriptive statistics (means and frequency counts) and bivariate statistics were run for the variables of interest and are presented in Table 1. The average age of the sample was 14 years old (SD = 1.99). The primary caregiver in the sample was a female, most often the biological mother (n = 303, 49.2%), followed by the grandmother (n = 180, 29.2%) or the aunt (n = 53, 8.6%). There was significant variation in the family member or relatives identified as the primary caregiver between orphans and non-orphans [*Χ*^*2*^(6) = 110.25, *p* < 0.00]. Orphans were more likely to be cared for by grandmothers followed by mothers. Aunts were more likely to be the primary caregivers of orphans than non-orphans. The majority of households were large in size, with about two thirds of caregivers caring for between one to two children or three to four children in addition to the child who participated in the study. The average age of caregivers in the sample was 48 years old (M = 48.58, SD = 13.85).

**Table 1:** Descriptive statistics of sample and variables of interest

	**Orphans**	**Non-orphans**	**Total**	**Statistical test**
**Adolescent characteristics:**				
Mean age (SD)	14.60 (1.94)	14.67 (2.02)	14.64 (1.99)	*t* (619) = 0.43, *p* = 0.67
Gender				
Male	93 (31.2%)	203 (68.8%)	298 (51.6%)	*χ*^2^ (1) = 0.03, *p* < 0.05
Female	112 (40%)	168 (60%)	280 (48.4%)	
Orphan status (%)	226 (36.3)	397 (63.7)	623 (100)	
Orphan type:				
Maternal (%)	76 (34.9)			
Paternal (%)	92 (42.2)			
Double (%)	50 (22.9)			
Household type:				
Orphan (%)			153 (24.6)	
Non orphan (%)			336 (53.9)	
Mixed (%)			134 (21.5)	
**Poverty related indicators:**				
Monthly income per capita (Poverty threshold = 37.5USD per capita per month)	$36.32 ($57.80)	$40.32 ($62.59)	$38.87 ($60.88)	*t*(599) = 0.77, *p* = 0.44
Monthly Household income (incl grants)	R1893.44 (R1672.76)	R1969.96 (R2128.10)	R1942.13 (R1973.54)	*t*(600) = 0.46, *p* = 0.65
**Household composition:**				
Total household inhabitants (M, SD)	7.83 (3.73)	7.37 (3.35)	7.54 (3.49)	*t* (620) = -1.59, *p* = 0.12
Number of adults in household (M, SD)	3.19 (1.82)	3.26 (1.82)	3.24 (1.18)	*t* (620) = 0.51, *p* = 0.61
Number of children in household	4.62 (2.53)	4.10 (2.20)	4.29 (2.34)	*t* (620) = -2.70, *p* <0.01
Dependency ratio (%)				
Care for child plus 1 – 2 more children	59 (28.9)	131 (36.1)	190 (33.5)	*χ*^2^ (3) = 6.70, *p* = 0.08
Care for child plus 3 – 4 more children	66 (32.4)	127 (35.0)	193 (34.0)	
Care for child plus 5 – 6 more children	47 (23.0)	68 (18.7)	115 (20.3)	
Care for child plus 7 or more children	32 (15.7)	37 (10.2)	69 (12.2)	
**Caregiver characteristics:**				
Primary caregiver				*χ*^*2*^(6) = 110.25, *p* < 0.00
Biological mother	52 (23.4%)	251 (63.7%)	303 (49.2%)	
Biological father	6 (2.7%)	22 (5.6%)	28 (4.5%)	
Aunt	33 (14.9%)	20 (5.1%)	53 (8.6%)	
Uncle	5 (2.3%)	2 (0.5%)	7 (1.1%)	
Grandmother	105 (47.3%)	75 (19.0%)	180 (29.2%)	
Grandfather	5 (2.3%)	7 (1.8%)	12 (1.9%)	
Other	16 (17.2%)	17 (4.3%)	33 (5.4%)	
Communication with caregivers about problems				*χ*^2^ (2) = 3.5, *p* = 0.17
Never	20 (9.3)	32 (8.1)	52 (8.5)	
Sometimes	83 (38.4)	125 (31.7)	208 (34.1)	
Always	113 (52.3)	237 (60.2)	350 (57.4)	
Caregiver impairment over the past month◊				*χ*^2^ (1) = 3.67, *p* = 0.06
Yes	128 (57.9)	196 (49.9)	324 (52.8)	
No	93 (42.1)	197 (50.1)	290 (47.2)	
Caregiver self-reported health				*χ*^2^ (3) = 4.45, *p* = 0.22
Poor	117 (52.7)	176 (44.7)	293 (47.6)	
Fair	25 (11.3)	50 (12.7)	75 (12.2)	
Good	68 (30.6)	135 (34.3)	203 (33.0)	
Excellent	12 (5.4)	33 (8.4)	45 (7.3)	
Caregiver provision of help and assistance				*χ*^2^ (2) = 4.72, *p* = 0.09
Never	9 (4.2)	8 (2.0)	17 (2.8)	
Sometimes	76 (35.3)	118 (30.0)	194 (31.9)	
Always	130 (60.5)	267 (67.9)	397 (65.3)	
**Psychosocial outcomes:**				
Anxiety/Depression	36.43 (9.80)	35.80 (10.07)	36.03 (9.96)	*t* (614) = -0.76, *p* = 0.45
Affability	35.86 (5.34)	36.54 (5.69)	36.30 (5.57)	*t* (618) = 1.46, *p* = 0.15
Resilience	23.47 (3.62)	23.56 (4.03)	23.53 (3.89)	*t* (272) = 0.27, *p* = 0.79

Note: missing values not included.

◊ This question item was phrased as, “during the past 30 days were you unable to perform your most important activity for an entire day because of problems with your physical or mental health?”.

Bivariate analyses showed that male and female participants did not differ significantly from one another in relation to anxiety/depression (*p* = 0.21), affability (*p* = 0.62) and resilience (*p* = 0.72). In addition, orphans and non-orphans did not differ significantly from one another in relation to any of the key variables of interest (poverty indicators, household characteristics, caregiver characteristics and child characteristics), including the psychosocial outcomes (resilience, affability and anxiety/depression). Orphan type (maternal, paternal or double orphans) was not associated with any differences in anxiety/depression [*F*(2, 212) = 0.74, *p* = 0.48], affability [*F*(2, 215) = 0.13, *p* = 0.88] or resilience [*F*(2, 215) = 0.17, *p* = 0.84]. The only significant difference that emerged from the bivariate analysis was that orphans tended to live in households (‘mixed’ as well as ‘orphan’ households) containing more children than those of ‘non-orphans’. Differences in caregiver impairment across orphan status just missed significance (p = 0.06), but suggest that caregiver impairment may be more common among caregivers of orphans rather than non-orphans in this sample.

### Multivariate analyses

Two way factorial ANOVAs were conducted to determine if the associations between the variables of interest and the three psychosocial outcomes differed by orphan status. The results are presented in Table 2. Generally, the associations between the independent variables of interest and psychosocial outcomes were the same for orphans and non-orphans. There were only two instances where orphan status significantly moderated the relationship between an independent variable and a particular psychosocial outcome. There was greater variation in affability across different levels of caregiver health among orphans compared to non-orphans. Furthermore, in cases where caregivers reported fair to good health, non-orphans’ affability scores were higher than those of orphans but, in cases where caregivers reported excellent health, orphans had substantially higher affability scores than non-orphans. There was a distinct pattern in the link between orphan status and perceived level of caregiver provision of help and assistance in relation to resilience. Non-orphans’ resilience scores were lower than those of orphans in cases where the children reported ‘little’ (i.e. “sometimes”) caregiver help and assistance and substantively lower in cases where children reported no (i.e. “never”) caregiver help and assistance.

**Table 2:** F tests of poverty, household, caregiver and child characteristics by orphans status on psychosocial outcomes

	**Anxiety/depression**		**Affability**		**Resilience**	
	**Orphans**	**Non orphans**	**Orphans**	**Non orphans**	**Orphans**	**Non orphans**
**Poverty related indicators:**						
Poverty threshold (threshold = 37.5USD per capita per month)^1^						
Households below poverty threshold	37.04 (9.79)	36.66 (10.36)	35.53 (5.46)	36.31 (5.80)	23.38 (3.73)	23.64 (4.08)
Households above poverty threshold	35.25 (9.67)	33.18 (8.91)	36.45 (4.86)	36.83 (5.81)	23.53 (3.41)	23.17 (4.11)
Monthly household income (incl grants) median = R1450						
Monthly household income below median of R1450	36.18 (10.00)	35.91 (10.14)	35.61 (5.69)	36.61 (5.63)	23.43 (3.71)	23.75 (3.93)
Monthly household income above median of R1450	36.77 (9.62)	35.29 (10.04)	36.11 (4.97)	36.42 (5.88)	23.36 (3.59)	23.30 (4.24)
**Household composition:**						
No. of inhabitants in household^2^						
1 – 4 people	36.16 (9.97)	33.20 (8.99)	35.98 (5.14)	36.83 (5.62)	23.12 (3.74)	23.51 (4.38)
5 - 7 people	34.97 (10.16)	35.12 (9.09)	36.15 (5.70)	36.61 (5.66)	24.08 (3.44)	23.70 (3.65)
8 – 10 people	37.06 (8.99)	37.61 (11.63)	34.72 (5.63)	36.79 (5.34)	23.19 (3.76)	23.65 (3.93)
More than 10 people	38.02 (10.05)	37.73 (9.92)	36.83 (4.47)	35.52 (6.39)	23.26 (3.52)	23.13 (4.62)
^3^Dependency ratio^4^						
1-3 children	35.02 (10.00)	34.56 (9.41)	35.70 (5.47)	36.72 (5.61)	23.24 (3.61)	23.64 (3.92)
4-5 children	35.83 (9.22)	35.80 (10.20)	35.86 (5.06)	36.95 (5.59)	23.75 (3.22)	23.76 (3.83)
6-7 children	36.55 (10.10)	37.40 (10.05)	36.51 (5.80)	37.30 (4.56)	23.55 (3.93)	23.78 (5.12)
More than 8 children	38.97 (9.57)	38.19 (10.14)	35.31 (4.88)	34.73 (6.82)	22.63 (3.97)	22.78 (5.12)
Number of adults in household^5^						
1-2 adults	36.35 (10.07)	35.17 (10.00)	35.69 (5.41)	36.40 (5.75)	23.41 (3.68)	23.39 (4.09)
3-4 adults	34.97 (9.22)	35.62 (9.78)	35.89 (5.78)	37.10 (5.36)	23.89 (3.68)	23.89 (3.70)
5 or more adults	38.78 (9.63)	37.30 (10.60)	36.08 (4.62)	35.93 (6.05)	22.94 (3.40)	23.35 (4.42)
Number of children in household^6^						
1 – 2 children	36.82 (10.26)	33.96 (9.31)	36.15 (5.24)	36.47 (5.75)	23.28 (3.47)	23.71 (4.20)
3-4 children	35.04 (9.01)	35.79 (9.78)	35.56 (5.53)	36.79 (5.53)	23.64 (3.45)	23.39 (3.67)
5-6 children	37.58 (10.55)	36.48 (10.30)	35.96 (4.69)	36.55 (5.82)	23.32 (3.72)	23.47 (3.80)
More than 7 children	37.17 (9.56)	38.12 (11.50)	35.83 (5.93)	35.85 (6.43)	23.48 (3.98)	23.04 (5.17)
**Caregiver characteristics:**						
Caregiver self-reported health^7,8^						
Poor health	36.67 (9.15)	36.82 (10.61)	36.12 (5.27)	35.89 (5.92)	23.74 (3.48)	23.17 (4.27)
Fair health	37.67 (9.14)	37.38 (10.00)	33.36 (4.38)	35.47 (5.24)	22.28 (3.02)	24.02 (3.06)
Good health	36.15 (10.44)	34.65 (9.52)	35.48 (5.46)	37.42 (5.40)	23.11 (3.88)	23.84 (4.09)
Excellent health	33.08 (11.73)	34.65 (9.53)	39.92 (4.81)	37.88 (5.62)	24.33 (4.21)	23.89 (3.77)
Caregiver impairment in last month^9^						
No	34.58 (9.10)	34.55 (9.43)	36.00 (4.74)	37.27 (5.62)	23.28 (3.37)	23.85 (4.03)
Yes	37.83 (9.86)	37.16 (10.53)	35.67 (5.76)	35.84 (5.69)	23.48 (3.79)	23.26 (4.02)
Communication with caregiver about problems^10^						
Never	39.75 (11.16)	40.50 (9.93)	33.05 (5.92)	33.72 (6.93)	23.50 (3.09)	21.47 (4.30)
Sometimes	36.71 (10.13)	36.38 (10.53)	35.06(5.49)	35.06 (5.98)	22.93 (4.00)	22.74 (4.32)
Always	35.11 (8.83)	34.91 (9.71)	37.31 (4.63)	37.66 (5.10)	24.07 (3.26)	24.26 (3.70)
Caregiver is helpful when in need of help or material things^11,12^						
Never	35.67 (6.25)	40.38 (10.84)	34.67 (5.29)	31.75 (7.46)	24.00 (2.74)	20.00 (6.00)
Sometimes	37.01 (10.06)	35.68 (10.44)	35.79 (5.73)	35.36 (5.83)	23.24 (4.022)	22.62 (4.19)
Always	35.69 (9.58)	35.67 (9.80)	36.28 (5.03)	37.14 (5.55)	23.78 (3.35)	24.05 (3.79)
**Adolescent characteristics:**						
Child age^13^						
10 – 12 years	34.52 (8.68)	34.27 (10.73)	35.71 (5.92)	37.44 (5.99)	23.11 (4.21)	23.79 (4.40)
13 – 15 years	35.61 (9.19)	35.46 (10.00)	36.45 (5.32)	37.23 (5.51)	23.32 (3.90)	23.62 (4.20)
16 – 18 years	38.21 (10.66)	36.72 (9.66)	35.20 (5.10)	35.31 (5.59)	23.81 (2.97)	23.45 (3.60)
Gender						
Males	38.08 (9.95)	36.10 (10.48)	35.84 (5.34)	36.56 (5.39)	23.27 (3.71)	23.68 (3.81)
Females	35.57 (10.03)	35.72 (9.36)	35.54 (5.25)	36.48 (6.07)	23.52 (3.55)	23.38 (4.36)

^1^Significant main effect for Poverty threshold on anxiety and depression scores, F(1, 580) = 7.78, p < .05.

^2^Significant main effect for number of household inhabitants on Anxiety and Depression scores, F(3, 607) = 3.45, p < .05.

^3^Number of children the caregiver cares for in the household.

^4^Significant main effect for Dependency ratio on Anxiety and Depression scores, F(3,554) = 2.67, p < .05.

^5^Significant main effect for Number of adults in household on Anxiety and Depression scores, F(2, 609) = 3.20, p < .05.

^6^This is the total number of children in the household irrespective of whoever cares for them.

^7^Significant interaction effect between caregivers’ self-reported health and orphan status and the DV Affability, F(3, 606) = 2.69, p < .05.

^8^Significant main effect for caregivers’ self-reported health status on adolescents’ affability scores, F(3, 606) = 5.06, p < .01.

^9^Significant main effect for caregiver impairment in the past month on adolescent’s anxiety and depression scores, F(1, 604) = 12.16, p < .01.

^10^Significant main effect for Communication with caregiver about problems and adolescents’ anxiety and depression scores, F(2, 599) = 6.14, p < .0, affability scores, F(2, 603) = 19.99, p < .01, and resilience scores, F(2, 604) = 9.40, p < .01.

^11^Significant main effect for caregiver provision of help and assistance on adolescents’ affability sores, F(2, 601) = 5.21, p < .01 and resilience scores, F(2, 602) =5.39, p < .01.

^12^Significant interaction effect between orphan status and caregiver provision of help and assistance on adolescents’ resilience scores, F(2, 602) = 3.02, p < .05.

^13^Significant main effect for adolescents’ age on depression and anxiety scores, F(2, 615) = 3.76, p < .05 and affability scores, F(2, 619) = 5.16, p < .05.

Table 2 also shows that the main variables of interest (household composition, poverty, caregiver and child characteristics) had significant direct effects on children’s psychosocial outcomes, irrespective of orphan status. Household composition was a significant factor in relation anxiety and depression reported by children. Larger households, larger numbers of adults in households and larger dependency ratios in households correlated with higher anxiety and depression scores for orphans and non-orphans. Similarly, living above or below the poverty threshold was significantly associated with anxiety and depression scores. Caregiver characteristics, most notably communication with caregivers about problems, correlated significantly with all three psychosocial outcomes. Caregiver impairment in the past 30 days was directly linked to higher child anxiety and depression and caregiver health was significantly associated with children’s affability scores. Children in late adolescence (16-18 years) reported higher anxiety and depression scores than children aged between 10 and 12 years (*p* < 0.05) and children between 13-15 years (*p* < 0.05). Children in late adolescence also reported lower affability scores than those aged 13-15 years (*p* < 0.01) and those aged between 10-12 years (*p* = 0.57). The gender of the child, however, was not statistically associated with their reported psychosocial wellbeing.

A hierarchical regression was conducted to determine the weighted effects of these independent variables (poverty threshold, household composition, caregiver characteristics and child characteristics) on participants’ anxiety/depression, affability and resilience scores. We did not run separate regression models for orphans and non-orphans in view of the results showing the relative insignificance of orphan status in relation to the independent variables in affecting psychosocial outcomes in children. The hierarchical regression models were also examined in terms of potential mediating and moderating relationships.

As illustrated in Table 3 all the models, although significant, explained very little variance in the three psychosocial outcomes as indicated by the R^2^ values. Standardised beta coefficients indicate that aspects of the caregiver-child relationship were the strongest predictors of psychosocial wellbeing among the child participants. Communication with caregiver about problems emerged as the strongest predictor of anxiety/depression, affability and resilience.

**Table 3:** Results for Hierarchical regressions run for three psychosocial outcomes

	**ANXIETY/DEPRESSION**		**AFFABILITY**		**RESILIENCE**	
	**Standardised beta coefficients (β)**		**Standardised beta coefficients (β)**		**Standardised beta coefficients (β)**	
	**When initially inserted**	**In final model**	**When initially inserted**	**In final model**	**When initially inserted**	**In final model**
**Adolescent characteristics (Block 1)**						
Age	0.08	0.03	-0.09	-0.03	0.02	0.06
Gender	-0.06	-0.01	0.00	-0.01	0.00	-0.00
Orphan status	0.11	-0.04	-0.07	-0.04	0.02	0.02
**Poverty indicators (Block 2)**						
Poverty threshold	-0.12**	0.07	0.04	-0.00	-0.06	-0.09
**Household composition (Block 3)**						
Dependency ratio	0.01	0.02	0.08	0.06	-0.05	-0.07
Total number household inhabitants	0.07	0.10	-0.09	-0.11	0.03	0.01
Total number of adults in household	-0.01	-0.02	0.02	0.05	-0.03	-0.00
**Caregiver characteristics (Block 4)**						
Communication with caregiver about problems		-0.15**		0.22**		0.16**
Caregiver provision of help and assistance		0.03		0.06		0.12*
Caregiver impairment in the past 30 days		0.12*		-0.12*		-0.08
Caregiver self-reported health		-0.11		0.03		0.00
R^2^		0.06		0.09		0.06

Anxiety/depression model: F = (11, 476) = 2.90**; Affability model: F = (11, 478) = 4.07**; Resilience model: F (11, 479) = 2.77**.

*p < .01, **p < .05.

In the anxiety/depression model, poverty threshold was significant after entry in its respective block but became non-significant in the final model where only communication with caregivers about problems and caregiver impairment were found to be significant predictors of anxiety/depression. In the affability model, communication with caregiver about problems and caregiver impairment were the only significant predictors of affability in the final model. In the resilience model, communication with caregiver about problems and caregiver provision of help and assistance accounted for a statistically significant proportion of the variance in resilience scores. Poverty threshold was very close to significance (*p* = 0.06) in the final model.

**Figure 1:**
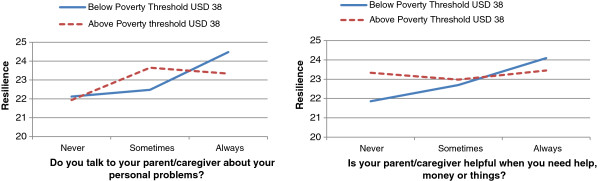
Poverty and caregiver characteristics interact in their influence on resilience scores.

Based on these results, moderating analyses were conducted to examine whether the influence of certain characteristics of the caregiver-child relationship on psychosocial outcomes differed in households living above and below the poverty line. Poverty threshold did not interact with communication with caregiver about problems (Interaction term: *p* = 0.31) nor with caregiver impairment (Interaction term: *p* = 0.83) to influence anxiety and depression scores. Poverty threshold, however, interacted with communication with caregiver about problems and resilience scores [*F*(2, 571) = 4.40, *p* < 0.05] as well as with caregiver provision of help and assistance and resilience scores [*F*(2, 576) = 3.14, *p* < 0.05] as is illustrated in Figure 1 respectively.

Although age of the child was directly related to anxiety/depression and affability in the ANOVA analyses, it was not significant in the regression models. Owing to this we wanted to explore whether the influence of age on children’s psychosocial outcomes was due to the level of communication with their caregivers about their problems. We felt that the association between participants’ age, communication about problems with caregivers and participants’ anxiety/depression and affability scores was more accurately analyzed as a possible mediating relationship. We used the method recommended by Baron and Kenny [32] and further, produced a Bootstrap analysis to derive a sampling distribution of the indirect effect (See [33,34]. The results in Table 4 show that communication with caregiver about problems was found to mediate the effect of child’s age on anxiety/depression scores and affability scores. That is, younger children reported more frequent communication with their caregivers about their problem which, in turn, was associated with a reduction in anxiety and depression and an increase in affability.

**Table 4:** Results of mediation analysis

**Mediation analysis**							
**Mediator: Communication with caregiver about problems (M)**	**Total Effect (IV → DV)**		**Direct effect (IV → DV when controlling for mediator)**		**Z scoreˠ**	**Lower CI**	**Upper CI**^**±**^
	**β**	**SE**	**β**	**SE**			
Child age (IV) **→** Anxiety/Depression (DV)	0.41*	0.20	0.29	0.20	2.64**	0.04	0.22
Child age (IV) **→** Affability (DV)	-0.29*	0.11	-0.16	0.11	-3.61**	-0.21	-0.06

Note: Only significant mediation analyses are shown in the table, *p < 0.05, **p < 0.01.

ˠResults of the Sobel test for indirect effects.

±Number of boostrap resamples = 1000.

## Discussion

This paper has examined the relationship between a set of pertinent factors categorized in terms of household characteristics, poverty, caregiver characteristics and child characteristics, on the psychosocial wellbeing of children living amidst an HIV epidemic. The purpose has been to contribute to debates on whether orphaned children are more prone to negative psychosocial outcomes than non-orphans. Our analysis of a data set from a relatively large cohort of children showed no significant differences in outcomes between orphans and non-orphans using three dimensions of psychosocial wellbeing (depression and anxiety, resilience and affability). Our analysis also showed very few significant differences in the effects of the range of social, psychological and material factors on orphans and non-orphans (e.g. orphaned children were more likely to be female and to live with more children in the household). These findings support recent research [13,14] which questions the primacy of orphanhood as a determinant of psychological outcomes in children living in high HIV prevalence settings.

There was a strong correlation between caregivers’ physical health, their caregiving capacities and children’s psychosocial wellbeing. Children with relatively high levels of anxiety and depression, irrespective of their orphan status, were those whose caregivers reported mental or physical impairment in the last month (*p* < 0.01). Children with lower affability were those whose caregivers reported poorer levels of health (*p* < 0.01).

Older children (between 16 -18 years) were more likely to have lower scores on affability (*p* < 0.01) and higher scores on anxiety/depression (*p* < 0.01) than children in lower age categories those below the age of 15. Of importance, the findings of the mediation analysis further suggest that more frequent communication with caregivers about personal problems and challenges may be responsible for younger children’s lower anxiety/depression and higher affability scores compared with their older counterparts. The latter finding highlights the protective role of caregiver-child communication in contexts of disadvantage and adversity [35].

There is a lot of research in sub-Saharan Africa highlighting the importance of the extended family as a safety net for orphans and, critically, the increasing burden, in terms of depletion of household resources and the strain on household resources resulting from accommodating more dependents [36-40]. Our results affirm quantitatively the findings in that literature. Household composition, whether it be larger households (*p* < 0.05), larger numbers of people in the household (*p* < 0.05) or larger dependency ratios (*p* < 0.05) had more deleterious effects on anxiety and depression scores than on the other two psychosocial outcomes irrespective of orphan status. The extended family remains a vital social security mechanism for care of orphans in the context of HIV/AIDS [41], yet the increasing size of extended families can bring with it an increasing likelihood of experiencing anxiety and depression among children and an increasing the risk of ill-health amongst caregivers. Accordingly, one must question assumptions about the capacity and capability of extended families to continuously absorb shocks to family welfare and recognize potential disadvantages for children as they enter adulthood.

While caregiver health was seen to be determinant in child psychosocial wellbeing, in the hierarchical regression analysis (see Table 3) it was evident that caregiver impairment rather than ill health was responsible for more deleterious effects on anxiety/depression (Caregiver impairment: β = 0.12, *p* < 0.01; caregiver health: -0.11, *p* > 0.05) and affability (Caregiver impairment: β = -0.12, *p* < 0.01; caregiver health: 0.03, *p* > 0.05). Given that the primary tasks or activities of the majority of caregivers were housework and child care, it is important to understand that the inability to fulfill the demands of caregiving and housework may be a more proximal predictor of poor psychosocial outcomes for children rather than the caregivers’ health status. In short, witnessing poor health and incapacitation in caregivers is stressful for children irrespective of whether they are orphans or not.

Orphan status was significant as a factor affecting psycho-social outcomes in two instances. First, the relationship between caregiver health and affability was significantly different for orphans and non-orphans (*p* < 0.05). Non-orphans had higher affability scores than orphans when their respective caregivers were in fair to good health but, in households where caregivers were in excellent health, orphans had more pronounced and higher affability scores than non-orphans (see Table 2). The intimation here is that orphans may require more emotional care than non-orphans, having experienced bereavement and change in living conditions [38,39]; hence, caregivers who are in ‘excellent’ health are more capable of meeting that need amongst orphans. Secondly, orphans’ resilience scores remain relatively stable across different levels of caregiver help and assistance (*p* < 0.05) but amongst non-orphans’, resilience scores are strongly, yet inversely, influenced by the level of help and assistance they receive from their caregivers (*p* < 0.05). Notably, orphans who reported not receiving any help or assistance from their caregivers had the highest resilience scores; higher than non-orphans and orphans who ‘sometimes’ and ‘always’ received help and assistance from caregivers. It appears that orphans’ resilience outcomes in this sample may be less reliant on interpersonal assets such as caregiver help and assistance, which could stem from the experience of losing parents and caregivers in the past. Hence, they may have learnt to rely on other assets – internal psychological assets or other interpersonal and social assets – to sustain their wellbeing after experiencing the loss of adult attachments in the past [42,43].

The extent to which the role of the caregiver could promote resilience in this sample of children was also found to vary according to the socio-economic status of the household (*p* < 0.05). Figure 1 illustrates that the role of the caregiver is a key resilience-promoting asset among children who were very poor. While low to moderate levels of caregiver help and assistance, including communication about personal problems, were associated with higher resilience scores for children who were not so poor, high levels of caregiver help and assistance, including communication about problems, were associated with better resilience outcomes for very poor participants. Furthermore, the wider variation in resilience scores across different levels of caregiver help and assistance and caregiver communication about problems for children who were very poor, suggests that their resilience scores are more strongly influenced by the level of support and assistance received from their caregivers. In accordance with Ungar’s theory on the ‘social ecology of resilience’, we can speculate perhaps that children living above the poverty threshold may have other social, material and interpersonal assets in their environment they can navigate towards to build resilience, but for adolescents living below the poverty line, who probably have fewer social and environmental resilience assets at their disposal, their relationship with their caregivers is a key resilience-promoting asset [44].

Indeed, being very poor was a significant contextual factor that was found to contribute to poorer mental health outcomes, in this study, higher anxiety/depression scores in children. Living below the poverty line can be associated with a myriad of stressors such as food insecurity, poor and inadequate housing, poor health, etc. all of which can thwart the satisfaction of adolescents’ basic needs and create worry and concern.

## Conclusions

The ACHWRP was designed to assess social effects of a marked and rapidly growing population of children orphaned as a result of the HIV epidemic in South Africa. The core question was whether the psychosocial and material welfare of orphans was significantly worse than non-orphans and hence, whether ‘AIDS orphans’ constituted a specific and significantly vulnerable category of children. The results of the project [28,45] generally show that orphans do not constitute such a category. In this paper we show that orphans did not report poorer psychosocial outcomes than non-orphans and did not differ significantly with regards to household, poverty and caregiver characteristics. This is not to deny the difficulties and stress experienced by the multitude of children who have witnessed the worsening health, followed by the loss of one or both of their parents. However, it is important to note that psychosocial outcomes among this sample of children were more strongly influenced by household composition/size, living above or below the poverty threshold and factors associated with the caregiver-child relationship and caregiver health. The results musters additional evidence for moving beyond narrow definitions of vulnerability towards a focus on orphans and vulnerable children, and more generally the consideration of the physical and psycho-social well-being of all children living in circumstances of poverty and HIV and AIDS.

This study carries several limitations. Firstly, our sample was school-based and by definition excluded households of children not enrolled in school. School attendance is seen as a protective factor; therefore children from in the latter households may have been more disadvantaged. Secondly, we only focused on selected variables for analysis in this study. Further studies would benefit from including important variables such as the extent and quality of peer relationships, perceived stigma and extent of social cohesion and level of belonging in family and school in impacting on child psychological wellbeing. Thirdly, the cross sectional nature of this study does not allow us to capture the dynamic and complex attribution pathways between orphan status, household characteristics, caregiver characteristics and child psychological wellbeing. As regards the latter, we have previously noted that the heightened burden placed on caregivers is associated with chronic illness, including depression [41]. Future research therefore needs to amplify the transactional relationship between caregiver physical and mental health and multiple child outcomes in impoverished contexts.

## Competing interests

The authors declare there are no competing interests.

## Authors’ contributions

KM conceptualised the manuscript, interpreted the data and assisted with drafting the manuscript. CR contribution was the statistical analysis and assistance in drafting the manuscript. TQ assisted in drafting the manuscript. GG conceptualised and assisted with drafting the manuscript. All authors read and approved the final manuscript.

## Pre-publication history

The pre-publication history for this paper can be accessed here:

http://www.biomedcentral.com/1471-2458/14/615/prepub
